# Identifying and synthesizing components of perinatal mental health peer support – a systematic review

**DOI:** 10.3389/fpsyt.2024.1389545

**Published:** 2024-06-20

**Authors:** Laura Hölzle, Philipp Schöch, Christine Hörtnagl, Anna Buchheim, Astrid Lampe, Ingrid Zechmeister-Koss, Jean Lillian Paul

**Affiliations:** ^1^ Department of Psychiatry, Medical University Innsbruck, Psychotherapy, Psychosomatics, and Medical Psychology, Division of Psychiatry I, Innsbruck, Austria; ^2^ University of Innsbruck, Institute of Psychology, Innsbruck, Austria; ^3^ Ludwig Boltzmann Gesellschaft Institute for Rehabilitation, Vienna, Austria; ^4^ Austrian Institute of Health Technology Assessment, Vienna, Austria

**Keywords:** peer support, social support, perinatal mental health, lived experience, parental mental illness

## Abstract

**Background:**

Becoming a parent, while often perceived as a joyous event, can also be a vulnerable life transition, with approximately one in five mothers experiencing perinatal mental illness. Peer support is recommended for its preventive and therapeutic benefits. However, relevant program components of perinatal mental health peer support remain to be identified.

**Objectives:**

This review aims to (1) identify peer support programs in perinatal mental health through existing reviews and to (2) synthesize the components of these programs.

**Methods:**

A systematic literature review guided by PRISMA was conducted searching four databases, supplemented by hand searches. The Template for Intervention Description and Replication (TIDieR) checklist facilitated the systematic extraction and synthesis of program components.

**Results:**

Eleven peer support programs were identified from three reviews, largely conducted in English-speaking countries. The identified reviews highlight the benefits of peer support in perinatal mental health. Key components of individual programs were contextual background, materials, provider training and support, delivery modes and locations, and evaluation. Sharing lived experience and providing flexible support were central to all programs.

**Conclusion:**

Aspects of flexibility, authenticity and the challenges of program evaluation in peer support must be considered. Findings can now inform future planning and implementation efforts of peer support programs in periantal mental health.

## 1 Introduction

Mental illness is the most common complication associated with pregnancy in Western countries, and affects approximately one in five mothers (1–3) and more than one in ten fathers (4–6) across the perinatal period, including one year after the birth of a child (7). A variety of risk factors are associated with perinatal mental illness (PMI) including a history of previous depression, low economic resources, isolation, lack of social or partner support, life stress, or marital dissatisfaction (8–10). Unintended pregnancy, past pregnancy losses (11, 12), first time mothers, traumatic events, or birth related factors can additionally increase the likelihood to develop a PMI (13). PMI in woman may lead to lower self-esteem, poor interpersonal relationships, higher levels of anger, an increased risk of mental illness in partners (14, 15), or suicidal behavior in severe cases (16, 17). It is well known that PMI also impacts infant development, including the child’s psychological adjustment (18–20). Without treatment, this can have significant effects on the mother, partner, infant, and wider family. Without treatment, PMI is also linked to economic consequences. For example, in the UK, the annual costs associated with a lack of timely access to perinatal mental healthcare have been calculated at the equivalent of nine billion euros with two thirds relating to long-term impacts on the child over the life course (2). Despite these individual and societal costs, many women do not access evidence-based care or receive effective treatment (21, 22).

Subsequently, it is necessary to enhance the prevention, screening, and treatment of PMI for new parents. Pharmacological therapies show mixed results and may be declined by women due to the potential harm to the fetus or negative effects on breastfeeding (23, 24). Non-pharmacological therapies in contrast have been found to be acceptable and help reduce perinatal symptoms e.g., counselling interventions, cognitive, behavioral, and interpersonal therapies (25, 26), psychosocial therapies (27), and interventions delivered by non-specialists or peers (12, 28, 29). Incorporating peer support in mental healthcare is being promoted by the World Health Organization (WHO) and is considered an essential component of mental health recovery, aligned with the WHO’s Convention on the Rights of Persons with Disabilities (30).

Peer support refers to the provision of social, emotional, and evaluative assistance by sharing similar lived experiences. The extent of support is based on the needs of the target population and can vary greatly. It can be provided through different modes of interaction e.g., individual sessions, self-help groups, or computer-mediated groups, and in diverse settings such as home, community organization, or via telephone (31). An underlying principle is that people who share similar experiences can offer a distinctive perspective (32), and better relate to each other with more authentic empathy and validation than what health professionals may be able to provide (33, 34). Peer programs have the potential to address the shortage of mental healthcare providers, especially in settings with low resources (35, 36). By shifting tasks to individuals with no formal training, gaps in perinatal mental health (PMH) service provision can be bridged, and in turn, improve access to services (37). Research has shown that peer support can increase levels of hope, empowerment, self-care, and decrease depressive symptoms (38). While distinct from therapy, peer support exhibits therapeutic elements. Peers connect over shared experiences, form connections and mutual support which reduce feelings of isolation and marginalization. Ultimately, peer support empowers individuals to look beyond diagnostic labels and envision a more meaningful and hopeful path forward (39). In addition, studies show beneficial effects for peer support workers themselves, including increased feelings of social worth and self-efficacy, through the experience of feeling valued by another individual (40, 41).

Originally, peer support has political roots, emerging from a civil rights movement. Rather than uniting over the shared experience of illness, as we know it today, peer support in the past connected individuals who have faced negative mental health treatment, emphasizing common reactions to issues such as coercion, overmedication, human rights violations, and a medicalized narrative (33). As a result, mental healthcare in many Western countries has shifted in the last decades to consider personal recovery and strength-based models. In Austria for example, a peer support movement was formally established in 2014 with the EX-IN (Experienced Involvement) training program which aims to train individuals with psychiatric diagnoses for roles in psychiatric and psychosocial services, fostering innovative, strength-based treatment approaches (42). A group of experts by experience advocate for the acknowledgement of peer support workers in mental healthcare. They propose the legal recognition and professionalization of peer support workers in Austria (43).

While most literature is conceptualized in a wider mental health setting, peer support research in PMH has mainly examined effectiveness (e.g., 29, 36, 44), and experiences with and impacts of peer support (e.g., 9, 45, 46). However, components of PMH peer support remain to be identified.

The aim of this paper is to systematically identify PMH peer support programs (see **Table 1**). Acknowledging the literature, this review draws on previous reviews to identify individual peer support programs and provides an overview of review characteristics (Part A). Ultimately, this paper synthesizes information on components of peer support programs (Part B). The findings of this study could lead to a comprehensive understanding of the design and implementation of PMH peer support, which can be utilized to inform practice development.

**Table 1:** Research aims and questions.

	Aim	Research Questions
Part A	To identify perinatal peer support programs in mental health within existing reviews	• Which reviews evaluate perinatal peer support programs in mental health and what are their characteristics?
Part B	To synthesize information on components of perinatal peer support programs in mental health	• What are details of the main components of perinatal peer support programs in mental health?

## 2 Methods

To address the research objectives, a systematic review guided by PRISMA guidelines was conducted (47). Prior to commencing the review, a detailed protocol was developed.

### 2.1 Literature search

In the first step, a search strategy was developed (see **Supplementary Material**) and used across academic databases, including PubMed, Web of Science, PsycInfo, and The Cochrane Database of Systematic Reviews, with which we could identify reviews and meta-analyses regarding PMH peer support. In the second step, the current review is informed by these identified reviews and meta-analyses as its primary source for identification of individual studies. This two-step process was complemented by hand-searching for reviews and single studies.

### 2.2 Inclusion and exclusion criteria

**Table 2** provides details on the process of inclusion and exclusion. Within the first step, we included reviews for full-text screening if they examined studies of peer support programs in mental health specifically targeting pregnant women or new mothers in the perinatal period. There were no time restrictions on results, and studies across all time periods until the search was conducted could be considered. Meta-reviews without single studies were excluded. Previous reviews were excluded if they included duplicate studies already covered by more recent and comprehensive reviews. This criterion was used to avoid redundancy. In the second step, we went through included reviews and identified relevant single papers for data extraction and further inclusion in this review paper. We included single papers if they involved peers with lived experience of PMI as peer support providers. We included doctoral theses and published project reports on peer support programs if they met inclusion criteria and were included in the selected reviews.

**Table 2:** Inclusion and exclusion process for reviews and single studies.

	Inclusion	Exclusion
Step 1: Reviews	Population: • Pregnant or postpartum women with PMI or at increased risk • Within perinatal period Study design: • Quantitative, qualitative, and mixed methods Other criteria: • Published in English peer-reviewed journals • From inception until August 2023	Other criteria: • Published in other languages • Meta-Reviews • Duplicate single studies
Step 2: Individual studies	Phenomenon of interest: • Peer support • Provided by peers with lived experience of PMI Study design: • Interventional and observational studies Outcome: • Description of program components	Phenomenon of interest: • Programs delivered by others than peers with lived experience of PMI Outcome: • No description of program components

### 2.3 Study selection

We identified 286 reviews and meta-analyses through the database search. After removing duplicates, the first (LH) and second (PS) authors independently reviewed titles and abstracts, followed by full-text eligibility screening. This process was repeated for the included reviews, covering 434 single studies. First and second authors assessed full-text single studies for eligibility, resolving conflicts through discussion or consultation with a third author (IZK).

### 2.4 Data extraction and analysis

For each review, information relevant to answering research questions from Part A was extracted (name of authors, year of publication, country, number of studies within review, research objectives, study design, and findings) and summarized narratively. Furthermore, single study characteristics (authors, year of publication, country, study type, publication type, target population, and primary aim of program) were extracted. For individual programs and to answer the research question from Part B, the Template for Intervention Description and Replication (TIDieR) framework was utilized to systematically extract program components. This tool was developed to improve the quality of intervention descriptions with the aim of simplifying reporting (48). It includes items related to the name, rationale, materials, procedures, providers, locations, mode and frequency of delivery, modifications and adherence to a particular intervention. The extracted information was uploaded and organized using QSR International NVivo Version 12 (49) and summarized narratively.

### 2.5 Quality appraisal

AMSTAR 2 (50) was chosen to assess the risk of bias of included reviews and is used for assessing randomized and non-randomized studies of healthcare interventions. The purpose of this quality appraisal is to gain an assessment of the risk of bias of the overall findings of the reviews addressing Part A. We did not assess individual studies within reviews, as they had already been assessed as part of the review in which they were included. Further, we used them to identify the components of peer support rather than the effectiveness of peer support.

## 3 Results

**Figure 1:**
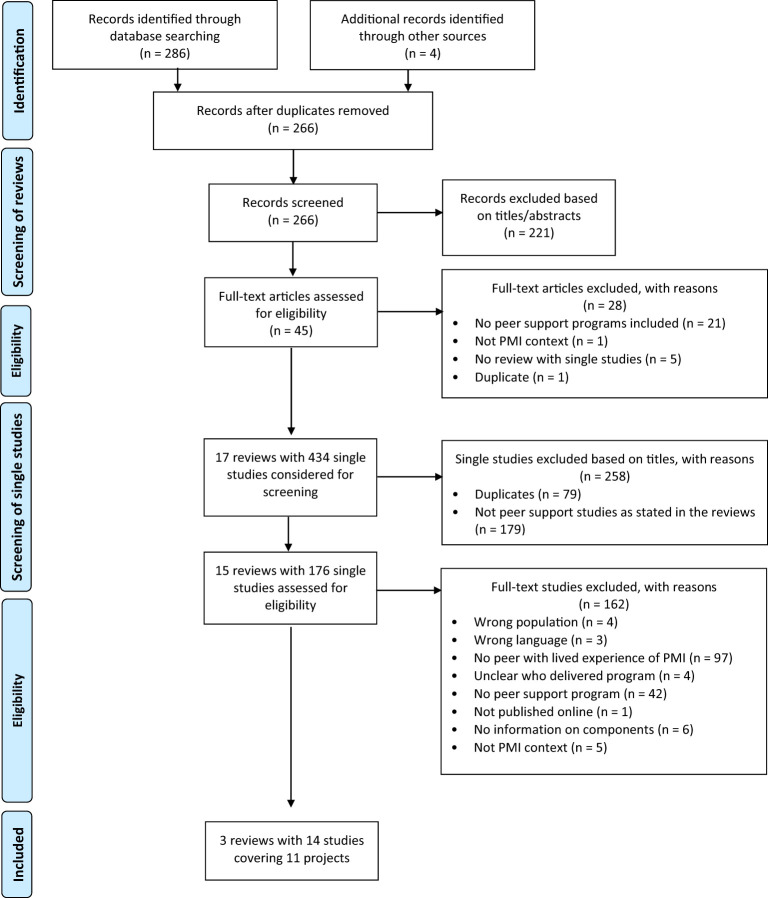
Modified PRISMA flow diagram based on (47).

The PRISMA flowchart (**Figure 1**) shows the selection process, whereby a total of three reviews and 14 individual studies were included in this review. The results are presented in two parts, providing an overview of reviews that evaluated individual PMH peer support programs and describe single study characteristics. In the second part, program components from individual studies are described and synthesized.

### 3.1 Characteristics of included reviews and single studies

Three selected reviews were published between 2020 and 2023 in China (n=2) and the United Kingdom (UK) (n=1). The reviews included a total of 54 studies. While the meta-analysis reviews focused on assessing the effectiveness of peer support on perinatal depression (29, 36), one review (51) aimed to explain how and why community-based PMH peer support works, using a realist review methodology. The latter included both qualitative and quantitative papers and provided a descriptive overview of study findings. They concluded that peer support works in various ways, influenced by personal and social contexts, with mostly positive outcomes. However, a culture of negativity, judging peers or experiencing peer support as stressful were identified as negative outcomes. Huang et al. (36) evaluated the effectiveness, cost-effectiveness, and satisfaction of PMH peer support interventions. Fang et al. (29) also examined mediating factors that could influence effectiveness such as timing, form, approach, frequency, and duration. Both meta-analyses suggested that peer support can be regarded as effective in reducing perinatal depressive symptoms (29, 36). Overall, the three reviews had a low risk of bias based on a quality assessment (see **Supplementary Material** for scoring and full data extraction tables).

**Table 3** provides an overview of included single studies. In subsequent sections, only program numbers are referred to, not study numbers. The programs were conducted in Canada [#1, 2, 4, 8, 11], the UK [#6, 7, 10], the United States (USA) [#3, 9], and Singapore [#5] between 2003 and 2019. These programs were evaluated with a variety of study types, including RCTs, qualitative, quantitative, and mixed methods, and publication types also including a doctoral thesis, and a protocol. Programs targeted mothers with postpartum depression up to nine months [#1–5] and two years postpartum [#10, 11]. Two programs did not specify the postpartum period [#6, 8, 9]. One program targeted pregnant women with antenatal depression [#7]. Most of the women were clinically at risk of postpartum depression and were screened using the EPDS [#1, 2, 4–6, 9–11], the Whooley questionnaire [#7], or the PHQ-9 [#3]. One program included postpartum women who self-reported postpartum depression [#8]. While most programs considered mothers aged 18 and over [#1, 2, 5, 8, 9], a few programs included mothers aged 16 [#3, 11] and 17 [4] onwards. Three programs did not specify the age of the participants [#6, 7, 10]. Where mentioned, exclusion criteria covered participants’ current use of antidepressant medication [#1, 2], history of psychiatric illness [#1, 5, 7], non-native English speaker [#6], or too mild or too severe depressive symptoms [#10]. While most programs aimed to decrease depressive symptomology [#1–6, 9–11], one program examined qualitatively how women talked about living through postpartum depression in the context of a peer support group [#8] and one program aimed to assess feasibility and effectiveness [#7].

**Table 3:** Included single studies.

# Program	# Study	Author, year	Country	Study type	Publication type	Identified through	Targeted population	Primary aim of program
1	1	Dennis, 2003b (52)	Canada	Quant (RCT)	Journal article	Huang et al., 2020 (36)	New mothers between eight and 12 weeks postpartum, 18y+, EPDS > 9	Decreasing postnatal depression
2	2	Dennis et al., 2009 (53)	Canada	Quant (RCT)	Journal article	Huang et al., 2020 (36)	New mothers two weeks postpartum or less, 18y+, EPDS > 9	Decreasing postnatal depression
	3	Dennis, 2010 (54)	Canada	Qual (Survey)	Journal article	Huang et al., 2020 (36)		
3	4	Gjerdingen et al. 2013 (55)	USA	Quant (RCT)	Journal article	Huang et al., 2020 (36)	Mothers with postpartum depressive symptoms with a 0-6 month-old infant, 16y+, PHQ-9 ≥10	Evaluating the benefit of postpartum doula support and peer telephone support
4	5	Letourneau et al., 2011 (56)	Canada	Quant (RCT)	Journal article	Huang et al., 2020 (36)	Mothers with infant less than 9 months of age, 17y+, EPDS >12	Evaluating home-based peer support that included maternal–infant interaction teaching
5	6	Shorey & Ng, 2019 (57)	Singapore	Quant (RCT)	Journal article	Fang et al., 2022 (29)	New mothers up to 3 months postpartum, 21y+, EPDS ≥9	Decreasing postnatal depression+anxiety, loneliness, and perceived social support
	7	Shorey & Ng, 2019 (58)	Singapore	Qual (Survey)	Journal article	(51)		
	8	Shorey et al., 2018 (59)	Singapore	Quan (Study protocol)	Protocol	Reference screening		
6	9	Cust, 2016 (60)	UK	Mixed methods (Pilot study)	Jouranal article (online)	McLeish et al., 2023 (51)	Mothers considered to be at elevated risk of perinatal depression, diagnosed by EPDS	Decreasing postnatal depression
7	10	Carter et al., 2019 (61)	UK	Mixed Methods (Feasibility study)	Journal article	McLeish et al., 2023 (51)	Pregnant women with antenatal depression, appr. 28–30 weeks gestation, using the Whooley questionnaire	Assessing the acceptability, recruitment, feasibility and effectiveness
8	11	Montgomery et al., 2012 (62)	Canada	Qual (Ethnography)	Journal article	McLeish et al., 2023 (51)	Postpartum women self-identified as having postpartum depression, 18-30y	Describing how women talked about living through postpartum depression in the context of a peer support group
9	12	Prevatt et al., 2018 (63)	USA	Mixed methods	Journal article	McLeish et al., 2023 (51)	Mothers with postpartum depression, 18y+, diagnosed by EPDS	Decreasing postnatal depression and evaluating participant satisfaction
10	13	Sembi, 2018 (64)	UK	Quant (RCT)	Doctroal thesis	McLeish et al., 2023 (51)	New mothers up to two years postpartum, >10 and <22 EPDS	Decreasing postnatal depression
11	14	Letourneau et al., 2015 (65)	Canada	Quant (Quasi experimental study)	Journal article	McLeish et al., 2023(51)	New mothers up to two years postpartum, 16-45y, EPDS 12-19	Decreasing postnatal depression

### 3.2 Synthesis of perinatal mental health peer support components

Program components based on the TIDieR checklist are described and synthesized. Consolidated results are presented in **Table 4**. A selection of components that are particularly relevant to answering the research question of Part B is presented in the text below.

**Table 4:** Full results of TIDieR framework.

Description*	Item
**Name or phrase which describes the peer support program**	**1. INTERVENTION NAME**
	• “Mothers helping mothers with postpartum depression” [#1] • “Peer (mother-to-mother) support” [#2] • “Technology-based peer-support intervention program (PIP)” [#5] • “Mums4Mums” [#10] • Not described [#3, 4, 6-9, 11]
**Contextual background and justification for the need for peer support in perinatal mental health**	**2. RATIONALE AND UNDERLYING THEORY**
	• Indicating high prevalence rates of PMI [#1, 2, 4, 5, 7-9] • Pointing out negative long-term outcomes for mothers/infants [#1-5, 7, 11] and partner/family [#5, 7] • Describing symptoms of PMI [#1, 4, 5, 6, 9, 11] • Outlining barriers for accessing help [#8, 9]/traditional treatments [#3, 4, 5, 11] • Highlighting positive impacts of social support on PMI [#1-7, 9-11] • Conceptualisation of peer support [#1, 2, 4, 7, 8, 11]
**Physical or informational materials used in the program, including those provided to mothers or used in program delivery or training of peer supporters**	**3. MATERIALS USED IN THE INTERVENTION**
	• Peer supporter training material ○ “Mothers Helping Mothers with Postpartum Depression” manual [#1, 2], adapted version of it [#10, 11] ○ Meeting guide (Canadian Mental Health Association, CMHA) 2009 [#8] ○ PPD brochure/resource list [#3] ○ (No name) Manual [#4]/Booklet [#5] ○ Not described [#6, 7, 9] • Peer supporter recruitment material ○ Flyers, advertisements in newspapers, word of mouth [#1, 2] ○ Nurses, study staff [#3] ○ From community [#4], personal invitation [#10] ○ Advertisements in local practices [#6, 7] and university [#7] ○ Emails [#5], online and offline advertisements [#11] ○ Not described [#8, 9] • Online blog for communication between research team and peer volunteers [#10]
**Any activities, other than providing peer support**	**4. PROCEDURES, ACTIVITIES AND PROCESSES**
	• Provision of supervision to peer supporters [#3-5, 6, 10] • Hiring additional staff [#1, 2, 4, 11] • Involvement of health professionals [#4, 9] • Advisory committee by people with lived experience [#8] • Informal gatherings [#10] • Special assessments of peer supporters [#4, 6, 10]
**Background and (duration/content of) training given to peer support providers**	**5. PROVIDER**
	• Background of provider: mothers with lived experience of PMI [#1-11] • Training duration ○ 4 hours [#1, 2]/half-day [#3, 5] ○ 8 hours [#4, 10, 11] ○ 2 days [#7] ○ Not described [#8, 9] • Structured training program [#1-5, 8-11] ○ Information on PMI [#3, 11], self-harm [#11], conducting appropriate referrals to professional services [#1, 2, 5, 11], clear understanding of peer role [#7, 10], how to provide informational, emotional, affirmational [#11] and practical support [#4, 5, 8, 9], telephone support skills [#1, 2, 5, 10, 11], group dynamics [#9], maternal-infant interactions [#4], active listening, behaviour change, and goal-setting [#10], and relationship building [#11] ○ Roleplay as a training strategy [#1, 2, 5] ○ No structured training program [#6, 7]
**Modes of delivery and whether it was provided individually or in a peer support group**	**6. MODE OF DELIVERY**
	• Individual telephone-based support [#1-3, 10, 11] • Individual home visits [#6, 7] + telephone-based support [#4] • Technology-based support via phone, messages, email [#5] • Group-based support [#8, 9]
**Types of locations where the program, peer supporter training and recruitment of mothers occurred**	**7. TYPES OF LOCATIONS**
	• Program location ○ Telephone/technology based [#1-5, 10, 11] ○ Mothers’ home environments/location of choice [#4, 6, 7] ○ Gynaecologist practice [#9] ○ Not described [#8] • Training location ○ Children centres [#10] ○ Not described [#1-9, 11] • Recruitment location of mothers with PMI ○ Local hospitals, clinics or health departments [#1-3, 5, 10, 11], web-based [#2], or phone-based screening [#11] ○ Not described [#8]
**Frequency and duration of peer support program and individual/groups sessions**	**8. FREQUENCY AND DURATION**
	• Duration of program ○ 4-6 weeks [#5-8] ○ Up to 12 weeks [#1, 3, 4, 11] ○ Between 24 weeks and 4 months [#2, 10] ○ Not described because ongoing program [#9] • Duration of single sessions ○ Tailored to mothers’ needs [#1-3, 5, 10, 11] ○ Weekly 1-2 hours sessions [#6-9] ○ Not described [#4]
**If the program was planned to be personalised, titrated or adapted: what, why, when, and how**	**9. TAILORING**
	• Tailoring in terms of contact frequency based on mothers’ needs [#1-3, 5, 10, 11] • Tailoring in terms of location based on mothers’ preferences [#4, 6, 7] • Not described [#8, 9]
**If the program was modified during the course of the study: what, why, when, and how**	**10. MODIFICATIONS**
	• No modifications reported [#1-11]
**If program adherence/fidelity was assessed: how and by whom**	**11. PLANNED ADHERENCE/FIDELITY**
	• Activity-logs [#1-7, 10] + field notes by professionals [#4] • Not described [#8, 9]
**If program adherence/fidelity was assessed: extent to which program was delivered as planned**	**12. ACTUAL ADHERENCE/FIDELITY**
	• Not described [#1-11]

*Description of item and how we applied it for the perinatal mental health peer support context.

### 3.3 Contextual background and justification

Rather than underlying theories, contextual background information and justifications for the need for peer support in PMH are provided. These include evidence from previous research, such as incidence rates of PMI [#1, 2, 4, 5, 7–9] and negative long-term outcomes for mothers and infants [#1–5, 7, 11] and the wider family [#5, 7]. Symptoms [#1, 4–6, 9, 11], such as low self-esteem, difficulties in coping, negative attitudes, feelings of inadequacy, loneliness, and risk factors for PMI [#1, 5, 6, 9, 11], such as inadequate social support and social isolation in particular [#6, 9, 11], are described. Barriers to traditional treatments [#3, 4, 5, 11] and help-seeking [#8, 9] are outlined justifying the need for peer support. These include concerns about medication interfering with breastfeeding [#3, 4], high treatment costs [#3, 4, 9], time constraints [#3, 9], and social stigma [#3, 5, 8, 9]. As a result, most programs emphasize the positive impact of social support on PMI [#1–7, 9–11], with some specifically highlighting the positive impact of peer support provided by peers with lived experience of PMI [#1, 2, 4, 6–8, 11]. One program also mentions the benefits of peer support for infant development [#4]. For the conceptualization of peer support, programs refer to Dennis (52) [#1], who defines peer support as “informational, appraisal (feedback), and emotional assistance” (p. 4) [#1, 2, 4, 7, 11].

### 3.4 Materials

The materials used in the programs focused on training [#1–5, 8, 10, 11] and recruitment [#6, 7, 9, 11] of peer supporters. Some programs used or adapted the “Mothers Helping Mothers with Postpartum Depression” manual [#1, 2, 10, 11] developed by Dennis (52) [#1], which outlines professional services and incorporates topics on how to provide effective telephone support. Another program developed a separate guide (66) [#8] intended to prepare group leaders to initiate and facilitate respectful support. Other training materials covered brochures and resource lists with contact information for support groups, classes, therapists, and other providers [#3]; a manual on four different types of support (informational, emotional, affirming, and practical) and how to teach optimal mother-child interactions [#4]; and a training booklet on referrals and skills required for technology-based support [#5]. Recruitment materials for peer supporters included various offline and online advertisements [#1, 2, 6, 7, 9–11].

### 3.5 Training and support for peer supporters

All peer supporters were community mothers with a lived experience of a PMI. Seven programs explicitly mentioned the need for recovery from PMI [#4–9, 11]. In the majority of programs, the peer supporters underwent structured training, ranging from four hours [#1, 2], half a day [#3, 5], eight hours [#4, 10, 11], to two days [#7]. Training content included information on PMI [#3, 11], self-harm [#1, 11], goal setting [#10], relationship building [#11], and making appropriate referrals to professional services [#1, 2, 5, 11]. Peers were supported to develop a clear understanding of their role [#7, 10] and to provide informational, emotional, affirming [#11], and practical support [#4, 5, 8, 9]. Training was adapted to suit the specific objectives, such as quality telephone support skills [#1, 2, 5, 10, 11], group dynamics [#9], or mother-child interactions [#4]. Role-playing was utilized as a training strategy [#1, 2, 5], while two programs had no structured training, but provided input on child protection procedures and confidentiality [#6, 7]. These two programs also emphasized the importance of providing organic support without receiving therapeutic training.

Additional activities described revolve around the peer supporter wellbeing, in terms of providing supervision to share experiences and discuss concerns [#3–5, 6, 10]. Other activities include the employment of peer coordinators to support the process of recruitment, matching, and program implementation [#1, 2, 4, 11]. Informal meetings for peer supporters were organized in one program [#10]. Three programs used an interview process and specific assessments to confirm the suitability of peer supporters [#4, 6, 10].

### 3.6 Delivery modes and locations

Few programs offered structured support, while most provided flexible, individualized support. Although training manuals were used, programs were flexible in terms of contact frequency [#1–3, 5, 7–11] or location [#4, 6, 7]. Sharing lived experience and providing support where deemed necessary were central to all programs. Individual telephone peer support was provided in five programs [#1–3, 10, 11]. Peer support home visits were delivered in two programs [#6, 7], one in combination with telephone calls [#4]. In the case of home visits, peer support was delivered in the mother’s home environment or in a place of their choice [#4, 6, 7]. One program was technology-based only, providing supportive telephone calls, emails, and text messages [#5]. While these programs were delivered on an individual one-to-one basis, two were delivered in groups [#8, 9]. One group-based support was delivered in a local waiting room of a gynaecologist practice [#9]. The programs recruited participants from local hospitals, clinics or health departments [#1–3, 5, 10, 11], used web-based screening [#2], or a telehealth service for screening [#11].

### 3.7 Evaluation of the programs

The planned procedures for monitoring fidelity were reported in eight programs that utilized peer-completed activity logs to examine peer-volunteer interactions [#1–7, 10]. One program also employed professionals to take field notes [#4]. While the programs provided details of the planned fidelity analysis, the extent to which the program was actually delivered was not described [#1–11].

## 4 Discussion

This review identified PMH peer support programs from three reviews. A brief overview of these reviews is also provided. The reviews were published in China and the UK between 2020 and 2023. The findings showed positive effects of perinatal peer support programs on mental health in the perinatal period. Eleven individual programs from these reviews were included in this study and published overwhelmingly in English-speaking areas (Canada, the USA, Singapore and the UK) between 2003 and 2019. Using the TIDieR framework, we reported details of key components. We synthesized and presented components related to contextual background, materials, support and training for peer supporters, delivery modes and locations, and evaluation of programs.

The first aim (Part A) was to identify PMH peer support programs within existing reviews and to provide an overview of the review findings which evaluated peer support programs in a PMH context. It was noted that different definitions of peer support providers were applied in the different reviews and studies included in the reviews. For example, peer support has been referred to as “social support as provided by another woman [ … ]” [(65), p. 3], by paraprofessionals (e.g., 57), pals (e.g., 67), non-specialists (e.g., 28), or unpaid volunteers (e.g., 55). Huang et al. (36) and Fang et al. (29) define peer support as being provided by mothers with significant similarities to the target population and personal experience of PMI, while McLeish et al. (51) expand the definition in their review to include one-to-one peer support, as well as peer support groups facilitated by non-peers. This suggests a lack of consensus regarding the definition of peer support, as also noted by Dennis (31) and Shalaby and Agyapong (68). Consequently, this contributes to a challenge in comparing studies and synthesizing evidence.

Despite small to moderate effects in reducing perinatal symptoms (29, 36) and potential drawbacks of peer support, such as a culture of negativity or experiencing peer support as a stressful social relationship (51), all included reviews emphasize the valuable benefits of peer support and its potential to prevent and treat PMI. This is consistent with previous reviews on PMH peer support that did not fulfil our inclusion criteria. Singla et al. (28) found evidence from high-income countries that peer support, delivered by non-specialists, can be effective in managing perinatal symptoms. Similarly, in a mixed-methods review analyzing interventions to prevent postnatal depression, Morrell et al. (44) identified peer support as one of the most beneficial interventions. Other qualitative analyses of reviews have identified similar challenges to peer support, such as time commitment and cultural differences as barriers (e.g., 65). However, the positive findings outweigh the negative ones. Recognition of the included reviews allowed us to take the second step of identifying individual programs.

Characteristics of individual programs demonstrate that the majority was published between 2003 and 2019 in mostly English-speaking areas (Canada, the USA, Singapore and the UK). This indicates a lack of recent evidence on peer support in PMH, and particularly in other regions. Apart from the three included programs from the UK, and other European studies that were conducted in the UK (e.g., 67, 69), it appears that research in Europe has mostly been conducted in the UK, potentially limiting the relevance of the findings to other European countries. PMH peer support may be different in countries such as Austria, which can be classified as traditional and conservative in relation to gender roles (70), and where, in rural areas, stigma and shame are associated with mental illness (71). There is a need to update and develop research in other countries to further understand contextual differences in PMH peer support.

The second aim (Part B) was to synthesize information on key components of peer support programs. Relevant components and details identified, based on the TIDieR framework, included contextual background, materials, support and training for peer support providers, delivery modes and locations, and evaluation of programs. Similar typologies were identified by Kotera et al. (72) in adult mental health, emphasizing recruitment, peer supporter preparation, practice, and peer supporter wellbeing. In particular, the success and sustainability of peer support work requires specialized recruitment strategies, robust training, regular supervision, and thoughtful peer matching, as highlighted by Moran (46). Nicholson and Valentine (73) underscore similar elements for parent peer support in mental health, including training, coaching, and support during implementation.

In terms of the materials used in the programs, most described training and recruitment materials. While some programs used or adapted a manual developed by Dennis (52), others developed their own materials. The diversity in the development of materials may indicate the adaptability and flexibility of PMH peer support programs to meet unique preferences. Flexibility has been identified as one of the ‘critical ingredients’ of peer support in mental health (33). Using resources that have already been developed and are available, as proposed by Leger and Letourneau (65), can provide a balance between flexibility and maintaining program consistency. Two programs made no reference to training materials, instead describing the provision of organic support (60, 61). This approach could be rooted in the value of authenticity in peer support and highlights the importance of avoiding overly intensive training sessions leading to professionalization of peer support (74). In contrast to this perspective, the EX-IN movement in Austria advocates for the professionalization of the peer support role in mental healthcare in terms of recognition by Austrian law with the establishment of a collective agreement. The debate between authenticity and professionalization in peer support echoes a tension between preserving the grassroots, experiential nature of peer support and integrating it into established healthcare structures (75). Various methods of recruiting peer supporters (e.g., flyers, advertisements, word of mouth) were documented. However, there is a gap in the literature as to the efficacy of these methods, leaving uncertainty about which approaches could be most successful.

Many programs documented the use of activity logs as a means of tracking fidelity, but there is a lack of detailed reporting on the actual fidelity across all programs. However, the focus of these programs may not be to evaluate fidelity or adherence but rather to assess program outcomes, impact, or effectiveness, which may explain the lack of specific details on fidelity. This may also be due to the challenges associated with evaluating a complex and adaptable program such as peer support. ‘Complex interventions’ are described as having multiple components, addressing different behaviors, requiring specific expertise from both providers and recipients, and allowing for some flexibility in the implementation. Such complexity therefore poses a challenge for the evaluation of such interventions (76). Traditionally, evaluations have focused on unbiased assessments of whether an intervention has achieved its intended outcomes. However, Skivington et al. (76) propose a new framework that broadens this focus. They emphasize understanding the overall impact, theorizing about mechanisms, considering the context of implementation, assessing contributions to systems change, and exploring practical uses of the evidence generated in real-world settings. This shift prioritizes the practical utility of information over mere effectiveness metrics. Additionally, an emphasis on stakeholder engagement in the evaluation of mental health services, including individuals receiving support, providers, and community members, ensures that diverse perspectives shape evaluation design (77). It is also considered an essential element in promoting patient-centered care (78). This holistic view on the evaluation of PMH peer support, however, is missing in current research.

### 4.1 Limitations

This review used the TIDieR framework to identify the main components of the programs examined. However, it is important to note that the programs were probably not originally designed to conform with the framework. As a result, the use of the framework in this context may compromise its validity for identifying the main components of these programs, which is a potential limitation to the reliability of the review’s findings. In addition, the adaptive nature of peer support, as described above, may not be easily captured or described within the structured framework provided by TIDieR. Attempting to force a flexible process into a structured framework may not accurately capture the essence of how peer support programs work in real-world settings. A further limitation lies in the application of the AMSTAR 2 tool for assessing the methodological quality of systematic reviews. This review provides an overview of three reviews rather than an assessment of effectiveness. Instead, the reviews served to identify individual studies and provide a narrative overview of the results of the reviews. Thus, some of the elements of AMSTAR 2 may not be directly applicable to this specific context. Additionally, PMH peer support programs without written documentation and currently existing programs without a published evaluation were not included in the search, potentially missing relevant programs. Future research will be crucial in developing a more comprehensive understanding of the specific formats and components that most effectively contribute to the success of PMH peer support initiatives.

## 5 Conclusion

This review identifies PMH peer support programs within previous reviews and provides review and study characteristics. Despite the lack of consensus on the definition of peer support in the literature, the included reviews highlight the benefits of peer support. Furthermore, this study synthesizes information on components of individual programs. Key components identified, based on the TIDieR framework, include contextual background, materials, support and training for peer supporters, delivery modes and locations, and evaluation. Despite the flexible nature of peer support programs in PMH, which supports the original principles of peer support, it also presents challenges for program evaluation. It also contrasts with recent debates about the professionalization of the role of peer support workers in mental healthcare. Further research in non-English speaking areas is warranted to fill existing gaps in the evidence base and to better understand contextual differences in PMH peer support. The findings outlined in this review provide valuable insights into program components and can now inform the planning and implementation of future PMH peer support programs.

## Data availability statement

The original contributions presented in the study are included in the article/**Supplementary Material**, further inquiries can be directed to the corresponding author.

## Author contributions

LH: Writing – review & editing, Writing – original draft, Visualization, Methodology, Investigation, Conceptualization. PS: Writing – review & editing, Validation, Conceptualization. CH: Writing – review & editing. AB: Writing – review & editing. AL: Writing – review & editing. IZ-K: Writing – review & editing, Supervision, Methodology, Conceptualization. JP: Writing – review & editing, Supervision, Methodology, Funding acquisition, Conceptualization.
