# Supplementary material for: Identifying and synthesizing components of perinatal mental health peer support – a systematic review
Source: Front Psychiatry. 2024 Jun 20;15:1389545. doi: 10.3389/fpsyt.2024.1389545 (PMC11223205; doi:10.3389/fpsyt.2024.1389545)
Supplement: Supplementary file 1 [file DataSheet_1.pdf]

![frontiers]

Supplementary Material

1 Supplementary Figures and Tables

1.1 Supplementary Figures

**Search term:**	("peer support" OR "social support" OR "self-help" OR "psychosocial interventions") AND ("perinatal mental health" OR "postpartum depression" OR "postnatal depression" OR "perinatal depression" OR "perinatal anxiety") AND ("systematic" OR "meta-analysis" OR "review") Filters: Meta-Analysis, Review, Systematic Review		
**Electronic databases searched**	**Date searched**	**Search type**	**Result**
PubMed	07.08.23	Title/Abstract	229
Web of Science	07.08.23	Title	16
PsycInfo	07.08.23	Title	27
CochraneDatabase of systematic reviews	16.08.23	Title/Abstract	14
Total			286
Total with hand searched reviews	07.08.23-16.08.23		289
Total after duplicates removed			**266**

Supplementary Figure 1. Search terms and results

1.2 Supplementary Tables

Supplementary Table 1. AMSTAR 2 assessment of included reviews

Items	Huang et al. (2020)	Fang et al. (2022)	McLeish et al. (2023)
1. Did the research questions and inclusion criteria for the review include the components of PICO?	+	+	+
2. Did the report of the review contain an explicit statement that the review methods were established prior to the conduct of the review and did the report justify any significant deviations from the protocol?	-	-	-
3. Did the review authors explain their selection of the study designs for inclusion in the review?	N.a.	N.a.	+
4. Did the review authors use a comprehensive literature search strategy?	+/-	+/-	+
5. Did the review authors perform study selection in duplicate?	-	+	-
6. Did the review authors perform data extraction in duplicate?	+	+	-
7. Did the review authors provide a list of excluded studies and justify the exclusions?	-	-	-
8. Did the review authors describe the included studies in adequate detail?	+	+	+
9. Did the review authors use a satisfactory technique for assessing the risk of bias (RoB) in individual studies that were included in the review?	+	+	+
10. Did the review authors report on the sources of funding for the studies included in the review?	-	-	-
11. If meta-analysis was performed did the review authors use appropriate methods for statistical combination of results?	+	+	N.a.
12. If meta-analysis was performed, did the review authors assess the potential impact of RoB in individual studies on the results of the meta-analysis or other evidence synthesis?	+	+/-	N.a.
13. Did the review authors account for RoB in individual studies when interpreting/ discussing the results of the review?	+	+/-	+
14. Did the review authors provide a satisfactory explanation for, and discussion of, any heterogeneity observed in the results of the review?	+	+	-
15. If they performed quantitative synthesis did the review authors carry out an adequate investigation of publication bias (small study bias) and discuss its likely impact on the results of the review?	+	+	N.a.
16. Did the review authors report any potential sources of conflict of interest, including any funding they received for conducting the review?	+	+	+

Supplementary Table 2. Characteristics of included reviews

Authors	Year	Country	No. of included studies	Objectives	Study design	Findings
Huang et al.	2020	China	5	To summarize evidence on effectiveness, economics, and satisfaction of peer support intervention on perinatal depression	Systematic review and meta-analysis	• Peer support interventions have a small-to-moderate reduction in perinatal depressive symptoms and is well receptive by pregnancy or postpartum women<br>• Peer support considered as cost-effective type to address the shortage of mental health care providers
Fang et al.	2022	China	1	To assess the effect of peer support on preventing or treating perinatal depression	Systematic review and meta-analysis	• Providing peer support during the perinatal period or only postpartum period, using Internet or telephone approaches, a combination of group and individual, at least once a week can be regarded as an effective measure to manage perinatal depression
McLeish et al.	2023	UK	5	To synthesise evidence on perinatal mental health peer support programmes outside mental health services by testing an initial theoretical model based on mechanisms, contexts and	Systematic realist review	• 13 C-M-O configurations explained take-up of peer support: peer support offers empathetic understanding and non-judgemental acceptance outside the social circle; relationships with

				outcomes (C-M-O framework), to understand what is it about community-based perinatal mental health peer support that works, for whom, in what circumstances, in what respects, and why		primary health professionals; cultural background and perspectives on mental health; desire for professional support; overcoming practical barriers; the format of the support; and the use of volunteers.<br>• 13 C-M-O configurations explained positive impact: empathetic listening, acceptance, affirmation and normalisation; peers sharing ideas about self-care, coping, and services; peers using therapeutic techniques; the opportunity to give support to others; meaningful social relationships with volunteers and other mothers; and other benefits of attending a group.<br>• 8 C-M-O configurations explaining negative impact: lack of validation; self-criticism from downward and upward social comparison; a culture of negativity; peers being judgemental or directive; not feeling heard; peer support as a

						stressful social relationship; and distress at endings.
